# Reproductive outcomes after non-occupational exposure to hexavalent chromium, Willits California, 1983-2014

**DOI:** 10.1186/s12940-017-0222-8

**Published:** 2017-03-06

**Authors:** Linda L Remy, Vera Byers, Ted Clay

**Affiliations:** 1 Family Health Outcomes Project, Family and Community Medicine, School of Medicine, University of California San Francisco, 500 Parnassus Ave. Room MU-337, San Francisco, CA 94143-0900 USA; 2 Immunology Inc, PO Box 4703, Incline Village, NV 89450 USA

**Keywords:** Hexavalent chromium (Cr(VI)), Non-occupational exposure, Domestic exposure, Reproductive health, Population health, Female, Pregnancy, Infant, Longitudinal research

## Abstract

**Background:**

From 1963–1995, a factory in Willits, Mendocino County, CA used toxic hexavalent chromium (Cr(VI)) without adequate measures to protect the population. We use longitudinal hospital data to compare reproductive outcomes for two generations in Willits and two generations in the Rest of County (ROC). This is the first study to quantify the reproductive impact of Cr(VI) in a non-occupational population.

**Methods:**

We searched California hospital discharge data (1983–2014) to find Mendocino County residents born 1950 or later. ZIP-code 95490 identifies Willits residents, with all others living in ROC. We used the Multi-Level Clinical Classification Software (CCS) to classify health outcomes.

First, we calculated the crude birth rate using an external census denominator. The next two models used self-contained denominators to assess health of infants and two generations of pregnant women. Finally, we focused on non-pregnant females and, for comparison, males. Here we added admissions for people who moved, linked and summarized admissions to the person level, and calculated rates per census population.

**Results:**

We found 29311 newborn records in ROC and 5036 from Willits. At start of period, Willits birth rate was low and did not recover until 12 years after Plant closure. While the Plant was open, respiratory conditions, perinatal jaundice, and birth defect rates were higher for Willits infants compared to ROC, but improved post-closure. ﻿Risk for abnormal birthweight and term was high and remained high over the study period.

During the period under study, we identified 31444 admissions of pregnant ROC women and 5558 from Willits. Willits women had significantly higher risk of pregnancy loss compared to ROC, whether stratified by generation, age group, or pre- and post-closure. Regardless of when exposed, Willits women continued to have significantly higher rates of in-hospital terminations, as animal studies of Cr(VI) exposure predict. In life course models, non-pregnant Willits women have significantly higher risk of reproductive organ conditions and neoplasms compared to ROC.

**Conclusions:**

Adverse reproductive outcomes are elevated and consistent with animal studies. General health outcomes reflect the same broad effect reported for occupationally exposed workers. For the first time, the detrimental reproductive effects of non-occupational Cr(VI) exposure in human females and their infants is reported.

**Electronic supplementary material:**

The online version of this article (doi:10.1186/s12940-017-0222-8) contains supplementary material, which is available to authorized users.

## Background

### History

The City of Willits, “Gateway to the Redwoods”, is in Mendocino County (the County), California. In 1950, a small auto shop expanded to a machine shop (the Plant) [1]. By 1959, the Plant manufactured heavy-duty steel cylinders [2] and, by the early 1960s, militarily-classified intercontinental ballistic missile cylinders [3, 4].

Steel was hardened with hexavalent chromium mist (Cr(VI)) which floated through vents in the Plant, polluting the air in Willits. At the top of the Eel River watershed, the Plant dumped toxic waste into local creeks and contaminated ground water at four sites in and near Willits [5]. After years of turmoil and investigations [6–11], the Plant declared bankruptcy, closing in late 1995 [12]. Years later, Agency for Toxic Substances and Disease Registry (ATSDR) investigators began to report increased risk of adverse health outcomes among Willits residents exposed to Cr(VI) emissions and ordered remediation to protect public health [13–18]. Remediation continues twenty years after closure [5].

In reports for ATSDR, Underwood and colleagues [14, 17] highlighted that few health studies address non-occupational exposure to environmental toxins, and that little is known about the long-term effect for men and women exposed during childhood or the reproductive period. Consistent with the Environmental Protection Agency (EPA) [19], they recognized that infants and children may be more sensitive to environmental exposures than adults, characterized risks to children in the Willits area, and felt information was needed on people who were children at the time of exposure. As a last point, they noted that research on the effect of Cr(VI) on children was an area with virtually no scientific information [17]. However, as exposure began so many years earlier, Harrison thought that too much time had elapsed to study outcomes [20].

These reports motivated Remy and Clay to use longitudinal hospital data to begin to assess health status for Willits [21]. After evaluating 1970 to 2000 census data, they concluded that Willits and the rest of the County (ROC) were among the more residentially stable areas in California and were demographically similar. Using hospital discharge abstracts over the period 1991-2012, census-based population denominators, and a cross-sequential (life course) design [22], they found that Willits residents had a significant increase in illness compared with ROC.

This paper continues the investigation, now focused on women born between 1950 and 1989, reproductive age (15 to 44) when first hospitalized for any reason between 1983 and 2014, and infants born over the same period. Primary data is hospital discharge abstracts, examining outcomes for infants, pregnant and non-pregnant women and, for comparison, men. Availability of this data allowed us to investigate the reproductive health of two generations of mothers and infants born to them. The generational focus allowed us to examine reproductive consequences for mothers exposed to Cr(VI) at different life stages and their babies. Most people in Willits were born and/or grew up when the air was contaminated. However, most of the older generation (born 1950–1969) were not exposed until puberty and conceived when the Plant was open (1983–1996), while most of the younger generation (born 1970–1989) were exposed in utero, during childhood, and conceived after closure (1997–2014).

### Hexavalent chromium (Cr(VI))

Although the Plant exposed Willits to various chemicals, we focus on Cr(VI) as the exposure of most concern for adverse outcomes. Cr(VI) attracted the primary interest of regulatory agencies that concluded past exposures affected community health [14]. This toxic makes its way into the domestic environment when facilities emit contaminated air, which then enters nearby water and soil. Principal non-occupational exposures occur by breathing contaminated air or drinking contaminated water.

A large body of research established serious reproductive toxicity in female and male animals and occupationally exposed men [23]. A major report summarized five studies of female animals exposed to Cr(VI) during gestation, and four of female animals exposed before reproductive age, with a focus on the resultant litters [24]. The first five studies had similar findings: increased pre- and post-implantation loss, resorption frequency, more dead and fewer fetuses per litter, low fetal weight, renal pelvis dilation, and bone or skeletal defects. In the second group of studies, exposure occurred long before mating. Results on litters were strikingly similar to litters of females exposed during gestation: decreased implantation, decrease in live fetuses, increased resorption frequency, and bone or skeletal defects. Others added more information on defects [25, 26] and reproductive organ damage [27–29].

Authors of one report [30] in the review felt that decreased numbers of implantation sites and viable fetuses, and increased resorption suggested disturbance of reproductive endocrine functions with multiple sites of toxicity along the hypothalamic-pituitary-ovarian uterine axis. They also suggested increased resorption was due to modification of the uterine lining before arrival of the embryo.

The number of corpora lutea also decreased [31–34]. Premature ovarian failure (POF) was noted when litters of exposed mothers reached reproductive age. Exposure during prenatal development causes POF in progeny by altering the expression pattern of certain enzymes in fetal ovaries [33, 35].

Whether exposed before or during gestation, most studies found an increase in chromium levels in the placenta, suggesting Cr(VI) accumulates in maternal tissues during treatment, remains during the untreated mating period, and crosses the placenta into fetuses during gestation [24]. In exposed male experimental animals and male humans, sperm viability, motility, and morphology is abnormal, and male workers report frequent spontaneous abortions in spouses [24, 36].

Only five studies describe adverse reproductive effects in occupationally exposed women. Similar to animal studies, these found: increased spontaneous abortions [37]; decreased intrauterine growth of fetuses resulting in low birth weight [38, 39]; high levels of Cr(VI) in the blood, urine, and umbilical cord blood [38, 40]; and in neonates, Cr(VI) in the cord blood and cord lymphocyte mutations [32, 41].

Here, we examine if similar outcomes occurred in a domestically exposed population of infants and pregnant women over the 32-year period 1983–2014, comparing women exposed during and after their reproductive years with those exposed only as children. We also examine the available life course data for non-pregnant women age 15 to 44 at some point in the 25-year period Jul-1990 through Dec-2014, when the oldest was about 64 years old. For comparison, we did a life course analysis for males.

## Methods

### Geographic descriptors

The term "County" describes the large, rural, sparsely populated area of Mendocino County. "Willits" (exposed) describes residents of ZIP-code 95490, which the County uses to report public health statistics [42]. The comparison is to residents in the rest of the County (ROC) (unexposed). From the 1970 census forward, these demographically disadvantaged populations have been among California’s more stable [21].

The 2010 census reported the following about these communities: County, population 87841, area 3506 square miles (3+ times larger than the State of Rhode Island); Willits ZIP-code 95490, population 13264, area 392 square miles; Willits proper (one of only four incorporated towns in the County), population 4888, area 2.8 square miles. The ZIP-code area enclosing Willits proper includes dispersed housing located in redwood wilderness, agricultural lands, and Native American rancherias.

### Population data

California releases county-level population estimates by age and sex annually, but sub-county population is available only every 10 years from the census. From the 1980, 1990, 2000, and 2010 censuses, we obtained county and ZIP-code population by age and sex [43–47]. ZIP-code was not available in 1980, so we used Tracts 106 and 107, which correspond with Willits ZIP 95490.

Within age and sex, we calculated the percent of Mendocino County population in Willits. We interpolated these percentages to obtain estimated percents for intercensal years, and extrapolated through 2014 using the 2000–2010 rates of change. For each age, sex, and year we multiplied the Willits percent times the county population estimate yielding a Willits estimate. We subtracted this from the county population to get ROC population. Finally, we approximated birth year by subtracting the age of the population estimated from the year of the estimate. For example, the population age 44 in 2014 was born in 1970. Summing annual populations within geography, sex and birth cohort yielded total person-years, used as the denominator to calculate crude birth rates and rates for life course models.

### Patient discharge data

We used 1983–2014 confidential patient discharge data (PDD) from California's Office of Statewide Health Planning and Development (OSHPD). These files were prepared previously for longitudinal research with methods described elsewhere [48]. Variables used include the patient’s birthdate, sex, ZIP-code, county of residence and admission, admission date, disposition, and principal and up to 24 secondary diagnoses (DX) and up to 20 secondary procedures (PX) classified based on the International Classification of Diseases, 9th Revision. In July 1990, California became the nation’s second state to include Social Security Numbers (encrypted to protect confidentiality (SSNC)) in discharge abstracts [49]. This key advance created the capacity to link together a person’s hospitalization history from 1990 forward and study outcomes over the life course.

To classify conditions, we used Level 1 (Body System) and Level 2 (Disease Condition) of the Multi-Level Clinical Classification Software (CCS) developed by the federal Agency for Healthcare Research and Quality (AHRQ) [50]. The CCS clusters diagnoses and procedures into a manageable number of clinically meaningful categories. Level 1 groups diagnoses (DXCH) and procedures (PXCH) by body system and is ideal for evaluating large aggregations of conditions. Level 2 groups diagnoses (DXCL) and procedures (PXCL) within a body system. Following methods used by the Healthcare Cost and Utilization Project [51], conditions were identified by searching all available fields. We classified birth defects using CCS defined groups, and identified others based on ICD-9 codes grouped by the Centers for Disease Control. See Additional file 1 for descriptions of groupers to select records and classify conditions.

From 1983 (first year data are available) through 2014 (last year available when we began this study), we extracted discharges with birth year 1950 forward, admitted while living in the County. We classified records geographically as Willits (ZIP-code 95490) or ROC (any other, with 11 missing ZIP-code). For life course models (non-pregnant females and males), we added admissions after people moved from the County. Data for life course models are available only from July 1990 forward when SSNC became available.

### Crude birth rates

We defined the number of births in Willits and ROC as the annual count of newborn discharges in the PDD. Population denominators were the total populations of these areas by year. We estimated crude birth rates (CBR) as the number of births per 1000 population. The CBR analysis used JoinPoint to fit piecewise linear models and to test for significant changes in slope between adjoining pieces [52, 53].

### Reproductive outcome files

We examined reproductive outcomes using hospital discharges of infants and pregnant women. Because newborn infants rarely have SSNC and mothers had them only since 1990, these were unlinked records of County residents from 1983–2014, identified at discharge as living in Willits or ROC. The infant file included newborn discharge records plus post-newborn records with age at admission less than one year. Post-newborn records are essential in picking up transfers and conditions often not noticed at birth. To check certain assumptions, a secondary analysis linked records for babies who were the only birth in the County on a given day. The pregnant women analysis file included any discharge record with a pregnancy diagnosis, age at discharge from 15 to 44 years, and year of birth in the interval 1950 to 1989.

Models for infants and pregnant women examined the rate of occurrence for various outcomes as a percent of discharge records, within Willits and ROC, with relative risk models comparing exposed (Willits) to unexposed (ROC) rates. In relative risk models, the rate numerator was the number of discharges with the outcome and the denominator was the number of newborn or delivery discharges, with rates rescaled per 10000.

For infants, we stratified by birth period as before (1983–1996) or after (1997–2014) Plant closure. We ended the pre-closure period in 1996 because the Plant closed in Dec-1995 [12] and parents conceived most 1996 births in 1995. For pregnant females, we stratified by 20-year generation (born 1950–1969, 1970–1989), age at pregnancy (15–24, 25–34, 35–44), and whether the pregnancy was before or after Plant closure. Results were so similar we focused on generation. Thus, the reported models focus on when people were born.

### Life course files

In life course (cross-sequential) models [22] for non-pregnant women and men, we define a person as the combination of SSNC, sex, and birth year. We include sex to identify a person because never-employed spouses can use their partner's SSNC [49]. For patients age 18–64, 95% of County admissions had SSNC with no statistically significant difference by sex or area, compared with 89% for California. Thus, we had a high likelihood of identifying adults no longer living in the County if admitted at least once while living there. In making these files, we excluded records lacking SSNC and SSNC with the same sex and more than one birth year or too many races or discharges, suggesting poor linkage.

We made life course files for non-pregnant females (and males reported tangentially) to assess general health, health of reproductive organs, and cancers. We assigned 20-year generation using birth year: 1950–1969 “Mothers/Fathers”, and 1970–1989 “Daughters/Sons”.

These models used discharges from age 15 through maximum age in 2014 (about 64), including discharges after people moved out-of-county. We first searched the recorded diagnoses and procedures on each record to flag conditions of interest. Summarizing records within person, a condition was set to 1 (true) if found on any record. We summarized person-level data by generation, divided summarized condition counts by external population estimates, and rescaled the resulting rate per 10,000 person years.

One County hospital did not use SSNC as part of its medical record system and we were unable to link records. Given this and other records without SSNC, we reduced the denominator person-years by the percent of in-county records with SSNC. This adjustment was within strata defined by 10-year birth cohort, sex, and Willits/ROC residence.

We assigned anyone who ever lived in Willits to Willits, and anyone who ever lived in ROC to ROC, ignoring admissions before the first in Willits for the Willits group and admissions before the first in ROC for the ROC group. To put the methodology in context, 52% of non-pregnant women had 1 discharge, 19% had 2, and 29% had 3 or more. Of 7057 women with more than one record, 529 lived in both areas. About 17% of non-pregnant women had post-County admissions, with no between-group difference.

### Statistical tests

When reporting results, we restrict use of the word *significant* to reflect statistical significance, specifically when the 2-tailed *P*-value is less than or equal to 0.05. Without stratification, chi-square tests assessed the statistical significance of relative risk (RR) with regard to exposure, with RR greater than 1 indicating greater risk in Willits. These models assessed data within a given interval (e.g., pre- or post-closure, 20-year generation, 10-year age group) or overall.

For stratified models, we used the Cochran-Mantel-Haenzel (CMH) test of RR. The CMH test allows for change of rate between strata, producing a combined estimate of RR. We report RR with lower (LCL) and upper (UCL) 95% confidence limits, obtained from the “Cohort Study” row of the “Estimates of the Common Relative Risk (Row1/Row2)” table produced by SAS Proc Freq. The Breslow-Day Chi-Square (BD) test compares RR between strata, that is, from one period to another, with the null hypothesis that RR stayed the same. Except for JoinPoint to analyse CBR trends, all programming was in SAS 9.4.

## Results

Table 1 shows, by 20-year generation and 10-year cohort within generation, potential exposure periods for people who ever lived in Willits. The table also shows number of records extracted for the four core models we developed: infant, pregnancy, non-pregnant female life course, and male life course.

**Table 1:** Number of records extracted by generation, exposure, model, and Plant period, 1983-2014

20-Year	10-Year	If Willits Resident			Model	Discharges		
		Exposed as:				Pre-closure	Post-closure	Row
Generation	Cohort	Ova	Child	Preg		1983-1996	1997-2014	Total
Mother/Father	1950-1959	No	Yes	Yes	Infant			
1950-1969					Pregnancy	5383	199	5582
					Female life course	7279	14527	21806
					Male life course	7221	14755	21976
	1960-1969	Yes	Yes	Yes	Infant			
					Pregnancy	9802	3152	12954
					Female life course	4204	9843	14047
					Male life course	4269	8090	12359
Daughter/Son	1970-1979	Yes	Yes	Some	Infant			
1970-1989					Pregnancy	4381	9853	14234
					Female life course	1730	5798	7528
					Male life course	1766	5100	6866
	1980-1989	Yes	Yes	Few	Infant	9101		9101
					Pregnancy	43	10265	10308
					Female life course	30	3569	3599
					Male life course	64	4202	4266
Grand child	1990-1999	70%	70%	No	Infant	8781	3510	12291
1990-2009	2000-2009	No	No	No	Infant		12224	12224
Great grand child	2010-2019	No	No	No	Infant		5656	5656
2010-2029								
Column Total					Infant	17882	21390	39272
					Pregnancy	19609	23469	43078
					Female life course	13243	33737	46980
					Male life course	13320	32147	45467

Table 1 addresses two important points. First, focusing on the column set labelled “If Willits Resident Exposed as”, each cohort within generation has slightly different exposure possibilities. Second, focusing on Pregnancy rows, only 199 admissions occurred post-closure among women born between 1950 and 1959, while all but 43 admissions occurred post-closure among women born between 1980 and 1989. This is the only birth interval containing both infants and parents.

**Fig. 1:**
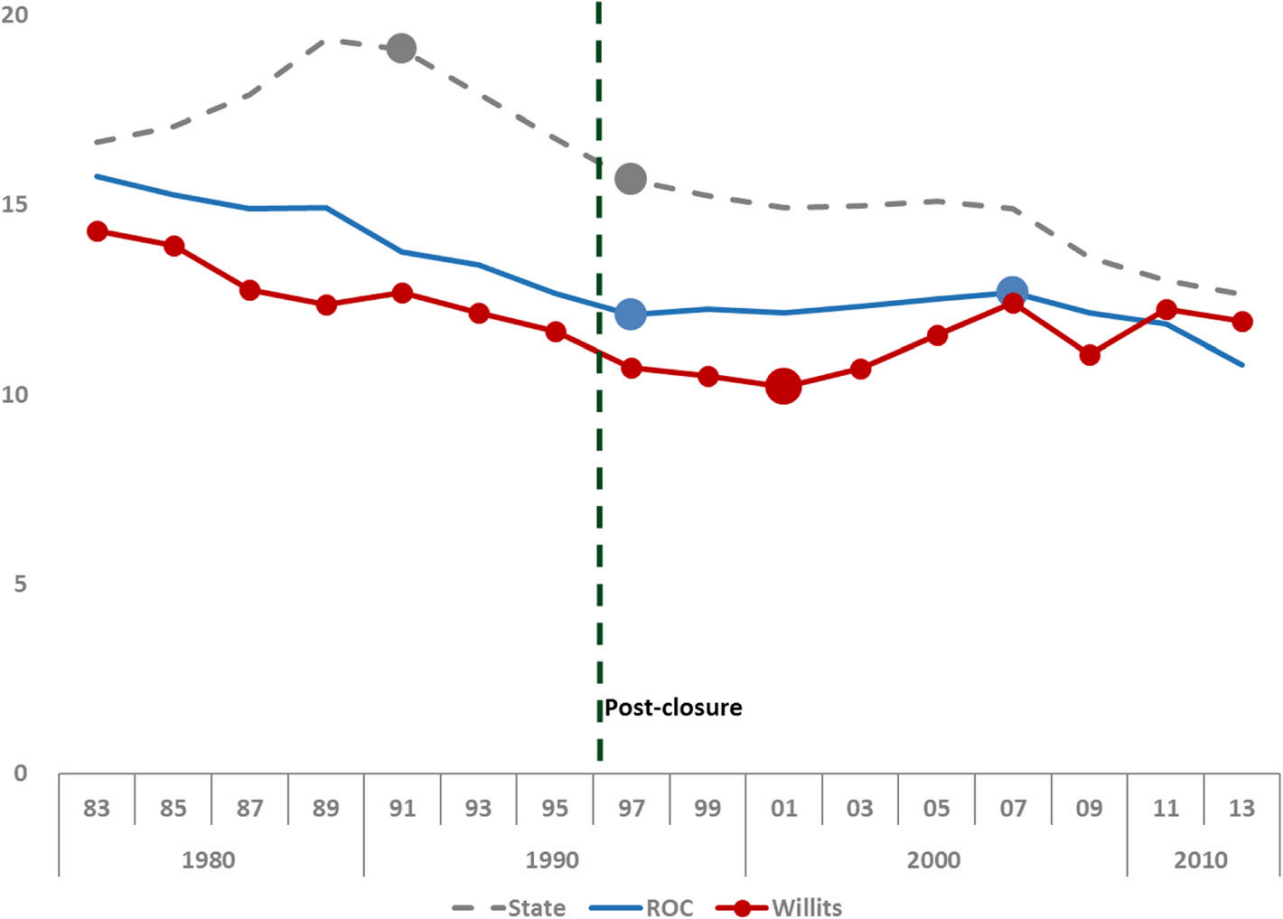
Births per 1000 population, California, ROC, Willits - 1983–2014

Fertility is a standard measure of population health, specifically the CBR per 1000 population. Figure 1 compares CBR trends over the period 1983–2014 for California, ROC, and Willits. The Plant began using Cr(VI) 20 years earlier, before data were available.

California trends changed in 1990 and 1997, while ROC trends changed in 1997 and 2008. By contrast, Willits CBR declined steadily through 2000 before beginning to increase. Inflection (or join) points have larger round circles. For the 3-year start-of-period (1983–1985), CBRs for both ROC and Willits were below the State, with Willits lower than ROC. By the 3-year end-of-period (2012–2014), the Willits CBR was not different from the State.

We next turned attention to infant outcomes. Between 1983 and 2014, we found 33505 discharges of ROC infants and 5767 of Willits infants age less than one year. These include post-natal admissions (ROC = 4194, Willits = 731) occurring after the newborn leaves the delivery hospital through age less than one year. ROC had 29311 newborn records and Willits had 5036.

Table 2 shows three groups of infant outcomes: conditions originating in the perinatal period, other general body system conditions, and congenital anomalies. The first column identifies the group and condition. Columns 2 and 3 show by residence the rate per 100 discharges (%) for the identified condition over the interval 1983–2014. Ignoring time and compared with ROC infants, bolded percentages indicate that Willits infants had significantly higher rates for the following: short gestation (preterm), low birth weight (LBW), or small for gestational age (SGA); macrosomia (large for gestational age); perinatal jaundice; and conditions affecting the nervous/sense system.

**Table 2:** Infant conditions by residence, Plant period, and adjusted by period, 1983-2014

Condition	Rate (%)		Relative Risk per Birth					Chi-sq P-Value	
	Discharges		Within Period		Adjust by Period				
	ROC	Willits	83-96	97-14	RR	LCL	UCL	CMH	BD
Total discharges	33505	5767							
Newborn	29311	5036							
Post-natal admissions	4194	731							
Origin in perinatal period									
Preterm, LBW, SGA	8.30	**9.43**	1.11	**1.16**	**1.14**	1.05	1.25	**0.0029**	0.5801
Macrosomia	5.63	**6.50**	**1.24**	1.12	**1.16**	1.04	1.29	**0.0058**	0.3909
Intrauterine hypoxia/asphyxia	4.35	4.27	1.10	**0.52**	0.97	0.85	1.10	0.6139	**0.0001**
Respiratory distress syndrome	1.58	1.56	0.96	1.01	0.99	0.79	1.23	0.9203	0.8410
Perinatal jaundice	13.96	**15.78**	**1.27**	1.07	**1.13**	1.06	1.20	**0.0002**	**0.0234**
General infant health									
General health condition (Any)	23.57	**25.11**	1.07	**1.08**	**1.08**	1.03	1.13	**0.0013**	0.5011
Infectious/parasitic	13.96	14.91	1.02	**1.10**	**1.09**	1.02	1.16	**0.0070**	0.4665
Endocr Nutri Metab Immun	2.01	1.96	0.94	1.00	0.98	0.80	1.19	0.8377	0.7761
Nervous/sense organs	5.19	**5.98**	1.11	**1.19**	**1.17**	1.05	1.31	**0.0059**	0.5950
Respiratory system	3.90	4.09	**1.29**	**0.82**	1.05	0.92	1.20	0.4942	**0.0012**
Digestive system	1.82	1.80	1.00	0.99	0.99	0.81	1.22	0.9587	0.9567
Genitourinary system	1.19	1.23	1.16	0.98	1.04	0.81	1.34	0.7500	0.5307
Congenital anomaly									
Genitourinary	1.22	0.99	1.28	**0.56**	0.81	0.62	1.07	0.1428	**0.0030**
Eye ear face neck cleft chrom	0.93	1.11	**1.59**	0.93	1.19	0.91	1.56	0.1963	**0.0489**

The next column set shows RR within period. Here, the RR reflects the condition rate for Willits divided by the condition rate for ROC. Within a period, a bolded number indicates that Willits infants had a significantly different RR than ROC infants. For example, in the pre-closure period 1983–1996, RR for Preterm/LBW/SGA was 1.11 for Willits infants compared with ROC, and post-closure RR was 1.16.

The next column set shows RR adjusted for residence and period. Newborn Willits infants consistently have been less likely to be normal birth weight. For example, risk for Willits’ newborn to be Preterm/LBW/SGA was high over the period (1.14, 1.05–1.25). That is, they were at about 14% increased risk (CMH *P* = 0.0029) with no significant change after closure (BD *P* = 0.5801). They also had overall 16% increased risk for macrosomia (1.16, 1.04–1.29, CMH *P* = 0.0085), significantly higher pre-closure (RR = 1.24), with no significant change post-closure (RR = 1.12, BD *P* = 0.3939). Risk for intrauterine hypoxia/asphyxia and perinatal jaundice dropped significantly post-closure.

The next group reports general infant health conditions, recorded by affected body system at birth or through the first year of life. Physicians recorded the presence of one or more conditions for about one-quarter of infant admissions. Risk for infectious/parasitic and nervous conditions was elevated over the interval and remained high relative to ROC with no significant change post-closure. Similar to newborn hypoxia/asphyxia, respiratory risk was high pre-closure, lower post-closure, reflecting a significant post-closure drop. Risk for digestive or genitourinary conditions was not elevated during infancy.

Turning to congenital anomalies, risk for genitourinary anomaly was non-significantly elevated pre-closure but dropped significantly post-closure. Risk for a sub-group of Other Congenital Anomalies -- eye, ear, face, neck, cleft, or chromosomal -- was significantly high pre-closure, with a significant drop post-closure.

Our next focus was on outcomes for pregnant women. Between 1983 and 2014, we found 31444 discharges of pregnant ROC women and 5558 of pregnant Willits women age 15 to 44. These include admissions that did not result in a live birth (ROC = 4395, Willits = 854). We used total records as the denominator to show the percent of pregnancy-related admissions by residence with the identified condition over the interval 1983–2014. ROC had 27049 delivery records and Willits had 4704, used as the denominator to calculate relative risk statistics.

We focused on three groups of variables: general health during pregnancy, conditions specific to pregnancy, and adverse pregnancy outcomes. Table 3 summarizes health status of pregnant women admitted to hospital by 20-year generation. As with infants, physicians recorded the presence of one or more general health conditions for about one-quarter of these admissions, with more health problems for pregnant women living in Willits (1.08, 1.03–1.13), and the younger generation (1970–1989) tending to be at greater general health risk than the older generation (1950–1969).

**Table 3:** Pregnancy conditions by residence, generation, and adjusted by generation, 1983-2014

Condition	Rate (%)		Relative Risk per Delivery					Chi-sq P-Value	
	Discharges		Within Generation		Adj by Generation				
	ROC	Willits	1950	1970	Ratio	LCL	UCL	CMH	BD
Pregnancy admissions	31444	5558							
Deliveries	27049	4704							
Non-delivery admissions	4395	854							
General health in pregnancy									
Body system condition (Any)	24.62	**26.11**	0.96	**1.12**	**1.08**	1.03	1.13	**0.0007**	**0.0026**
Infectious/parasitic	9.94	**10.94**	**1.24**	**1.11**	**1.13**	1.04	1.22	**0.0033**	0.5211
Endocr Nutri Metab Immun	3.65	4.12	1.02	**1.19**	**1.15**	1.00	1.32	**0.0434**	0.3686
Blood/blood-forming organs	5.07	4.35	0.90	0.87	**0.88**	0.77	1.00	**0.0476**	0.8029
Mental disorders	4.64	**6.42**	1.06	**1.53**	**1.41**	1.26	1.58	**0.0000**	**0.0082**
Nervous system sense	1.20	**1.53**	**0.54**	**1.64**	**1.30**	1.03	1.64	**0.0281**	**0.0007**
Circulatory system	1.57	1.71	0.82	1.19	1.11	0.89	1.38	0.3499	0.2037
Respiratory system	2.11	**2.68**	1.05	1.39	**1.30**	1.09	1.54	**0.0037**	0.1854
Digestive system	2.32	2.30	0.73	1.15	1.01	0.84	1.22	0.9037	**0.0353**
Genitourinary system	3.32	3.65	1.00	1.18	1.12	0.97	1.30	0.1282	0.3181
Pregnancy, birth, puerperium									
Hemorrhage in pregnancy	2.28	1.93	0.84	0.88	0.86	0.70	1.05	0.1342	0.8023
Hypertension in pregnancy	5.92	6.23	1.06	1.08	1.07	0.96	1.20	0.2248	0.8541
Early labor	9.97	10.67	1.09	1.09	**1.09**	1.00	1.18	**0.0451**	0.9549
Long pregnancy	7.25	6.71	1.06	0.92	0.95	0.85	1.05	0.2907	0.2508
Diabetes mellitus in pregnancy	5.64	**4.28**	0.94	**0.71**	**0.77**	0.68	0.88	**0.0001**	**0.0383**
Malposition	6.04	6.12	1.07	1.00	1.03	0.92	1.15	0.5937	0.5094
Pelvic obstruction	5.21	4.84	0.87	1.02	0.94	0.83	1.07	0.3675	0.2110
Previous Cesarean-section	10.37	10.04	1.01	0.96	0.98	0.91	1.07	0.7226	0.5905
Fetal distress	17.89	17.27	1.03	0.94	0.98	0.92	1.04	0.5634	0.1395
Premature rupture amniotic fluid	4.56	4.28	0.95	0.96	0.95	0.84	1.09	0.4954	0.8935
Hydramnios	2.04	1.53	**0.55**	0.87	**0.76**	0.61	0.95	**0.0170**	0.0827
Umbilical cord	17.87	18.01	**1.12**	0.98	1.03	0.97	1.09	0.3750	**0.0311**
Other: Reproductive anomaly	2.26	2.28	0.98	1.06	1.03	0.86	1.24	0.7518	0.6947

As to conditions specific to pregnancy, birth, and the puerperium, the finding of increased risk for Willits women to have early labor is consistent with patterns in infants for preterm birth. Other pregnancy indicators tend to indicate normal or low risk for Willits.

A successful pregnancy results in one admission with the birth of a live baby while admission that does not result in a live birth is high-risk. Table 4 stratifies admissions not ending in live birth into two mutually exclusive groups: those with return home still pregnant (10.5%), and those without a live birth (pregnancy termination, 2.9%), with three main termination outcomes listed separately: ectopic pregnancy, spontaneous abortion, and pregnancy termination procedure.

**Table 4:** Non-delivery pregnancy events per 10,000 deliveries by time measure, period and residence, 1983-2014

Outcome	Time		Residence			Adj Rel Risk			Chi-sq P-Value	
	Measure	Period	ROC	Willits	RR	Ratio	LCL	UCL	CMH	BD
Return home still	Generation	1950-1969	1273	1461	**1.15**					
Pregnant		1970-1989	1075	1027	0.96	1.05	0.97	1.14	0.2614	**0.0310**
	Plant	1983-1996	1350	1541	**1.14**					
		1997-2014	1005	930	0.93	1.04	0.96	1.13	0.3173	**0.0144**
Pregnancy termination	Generation	1950-1969	433	539	**1.25**					
		1970-1989	223	303	**1.36**	**1.29**	1.11	1.50	**0.0011**	0.6197
	Plant	1983-1996	400	488	**1.22**					
		1997-2014	245	336	**1.37**	**1.28**	1.10	1.50	**0.0014**	0.4798
Ectopic pregnancy	Generation	1950-1969	209	200	0.96					
		1970-1989	77	126	**1.63**	1.17	0.92	1.50	0.2081	**0.0369**
	Plant	1983-1996	199	193	0.97					
		1997-2014	82	129	**1.57**	1.16	0.91	1.48	0.2420	0.0566
Spontaneous abortion	Generation	1950-1969	51	91	**1.78**					
		1970-1989	25	46	**1.83**	**1.80**	1.20	2.68	**0.0038**	0.9490
	Plant	1983-1996	45	85	**1.88**					
		1997-2014	29	49	1.66	**1.79**	1.19	2.67	**0.0042**	0.7703
Therapeutic D&C	Generation	1950-1969	141	234	**1.66**					
		1970-1989	90	111	1.23	**1.47**	1.15	1.88	**0.0020**	0.2449
	Plant	1983-1996	135	233	**1.72**					
		1997-2014	93	105	1.13	**1.46**	1.14	1.87	**0.0024**	0.1080

Whether analysed by generation or pre/post closure, adverse pregnancy outcomes were more common for Willits women than ROC women with similar results no matter how stratified. The earlier, pre-closure group of pregnant Willits women had higher risk of returning home still pregnant than their ROC peers, with post-closure Willits women at normal risk of returning home still pregnant.

Both generations of Willits women had significant increased RR for pregnancy termination and sub-conditions. Additionally, the 1960–1979 generation had high RR for ectopic pregnancy and the 1950–1959 generation for termination procedures. We also stratified by age group, with similar results.

Our last focus was long-term life course outcomes. Specifically, for the 25-year period Jul-1990 to Dec-2014, Table 5 shows results of the life course analysis summarizing 30954 non-pregnant discharges for 11150 women ever living in ROC, and 6700 for 2553 women ever living in Willits. For comparison, Table 6 shows life course results for males, summarizing 29556 discharges for 10997 men ever living in ROC and 6094 for 2274 men ever living in Willits.

**Table 5:** Non-pregnant female health by place, generation, and adjusted by generation, life-course 1990-2014

Condition	Rate (%)		Relative Risk per Population					Chi-sq P-Value	
	People		Within Generation		Adj by Generation				
	ROC	Willits	1950	1970	Ratio	LCL	UCL	CMH	BD
Illness burden									
People	11150	2553	**1.16**	**1.25**	**1.19**	1.14	1.24	**0.0000**	0.1234
Discharges	30954	6700	**1.10**	**1.19**	**1.12**	1.10	1.15	**0.0000**	**0.0095**
General health									
Infectious/parasitic	24.33	24.44	**1.23**	1.10	**1.19**	1.09	1.30	**0.0001**	0.2663
Endocr Nutri Metab Immun	46.94	47.36	**1.19**	**1.23**	**1.20**	1.13	1.28	**0.0000**	0.6632
Mental disorders	52.83	**56.48**	**1.26**	**1.28**	**1.27**	1.20	1.34	**0.0000**	0.8635
Nervous system sense organs	28.88	**32.67**	**1.33**	**1.37**	**1.34**	1.25	1.45	**0.0000**	0.7437
Circulatory system	34.39	**36.51**	**1.24**	**1.37**	**1.26**	1.18	1.36	**0.0000**	0.2785
Respiratory system	30.45	**34.04**	**1.31**	**1.37**	**1.33**	1.23	1.43	**0.0000**	0.5789
Digestive system	45.70	**50.76**	**1.31**	**1.35**	**1.32**	1.24	1.40	**0.0000**	0.6283
Reproductive health									
Neoplasm	24.76	24.83	**1.16**	**1.42**	**1.20**	1.10	1.30	**0.0000**	0.0933
Reproductive organ	6.28	6.70	**1.27**	1.27	**1.27**	1.08	1.50	**0.0046**	0.9961
Benign	15.87	15.08	1.11	1.32	**1.13**	1.02	1.27	**0.0254**	0.2725
Genitourinary system	46.30	47.12	**1.17**	**1.34**	**1.21**	1.14	1.29	**0.0000**	0.0571
Pelvic inflammatory disease	13.61	**15.04**	**1.28**	**1.42**	**1.31**	1.17	1.47	**0.0000**	0.4313
Endometriosis	11.28	11.32	**1.18**	1.26	**1.19**	1.05	1.36	**0.0064**	0.6768
Menstrual disorder	14.91	14.45	1.07	**1.44**	**1.15**	1.03	1.29	**0.0129**	**0.0252**
Ovarian cyst	7.46	**8.97**	**1.38**	**1.53**	**1.43**	1.23	1.65	**0.0000**	0.5278
Reproductive organs (PX)	6.57	**5.37**	**1.09**	**1.41**	**1.15**	1.06	1.25	**0.0006**	**0.0086**

**Table 6:** Male health by place, generation, and adjusted by generation, life-course 1990-2014

Condition	Rate (%)		Relative Risk per Population					Chi-sq P-Value	
	People		Within Generation		Adj by Generation				
	ROC	Willits	1950	1970	Ratio	LCL	UCL	CMH	BD
Illness burden									
People (N)	10997	2274	**1.16**	**1.20**	**1.17**	1.12	1.23	**0.0000**	0.4643
Discharges (N)	29556	6094	**1.15**	**1.20**	**1.16**	1.13	1.20	**0.0000**	0.2952
General health									
Infectious/parasitic	26.60	28.50	**1.22**	1.37	**1.25**	1.15	1.37	**0.0000**	0.2481
Endocr Nutri Metab Immun	41.47	43.00	**1.21**	**1.22**	**1.21**	1.13	1.30	**0.0000**	0.9556
Mental disorders	58.04	58.57	**1.18**	**1.20**	**1.18**	1.11	1.26	**0.0000**	0.7732
Nervous system sense organs	27.87	**32.38**	**1.32**	**1.50**	**1.36**	1.25	1.48	**0.0000**	0.1803
Circulatory system	39.28	41.15	**1.19**	**1.41**	**1.23**	1.14	1.32	**0.0000**	0.0912
Respiratory system	31.39	**33.81**	**1.23**	**1.35**	**1.26**	1.16	1.37	**0.0000**	0.3461
Digestive system	41.25	**44.67**	**1.24**	**1.36**	**1.27**	1.18	1.36	**0.0000**	0.2280
Reproductive health									
Neoplasm	9.93	10.25	1.16	**1.50**	**1.21**	1.04	1.40	**0.0107**	0.2163
Genitourinary system	19.84	**21.66**	**1.22**	**1.51**	**1.28**	1.16	1.41	**0.0000**	0.0933

Reflecting community burden of illness, physicians admitted more Willits women and men per population and they had more discharges per person. At an earlier life stage, RR for younger Willits residents is equivalent to older Willits residents. Conditions elevated for Willits residents at birth or pregnancy are elevated over the life course.

With the oldest not yet age 65, Willits women and men had increased risk for any neoplasm and genitourinary conditions. Willits women had higher risk for reproductive and benign neoplasms and for reproduction-related conditions: pelvic inflammatory disease, endometriosis, menstrual disorders, ovarian cysts, and reproductive organ surgeries.

## Discussion

Cr(VI) causes DNA strand breaks, chromium-DNA adducts, DNA protein cross links, and oxidative DNA damage [54–58], damaging mammalian cells by binding to and distorting DNA, causing aberrant expression. Helped by nonspecific anion carriers, Cr(VI) crosses the cellular membrane, links with oxygen, and is a strong oxidizing agent. Intracellular reduction of Cr(VI) to Cr(III) leads to extensive formation of DNA-phosphate-based adducts, causing genetic damage [59]. As a result, Cr(VI) is toxic, carcinogenic, mutagenic, and teratogenic.

In this context, we organize our findings in two major sections: the impact of non-occupational Cr(VI) exposure on reproductive health and general health. We close with a discussion of the study’s strengths and weaknesses.

This study focused primarily on reproductive problems. Both animal and human occupational studies confirm that exposing a pregnant female to a teratogenic chemical will harm the pregnancy. Animal studies also indicate that exposing a female before reproductive age is as harmful as exposure in pregnancy. A primary purpose of this study was to determine if similar harm occurs in domestically exposed women. We focused on pregnancy outcomes and the health of two generations of mothers and babies.

A critical concept underlies our work: generational change. As Table 1 showed, except for people arriving after Plant closure, the entire population of Willits was exposed to Cr(VI) before closure. Of the older generation (1950–1969, 78% of pre-closure pregnancy admissions), most were exposed during reproductive years. Given later exposure, reproductive organs of many in the 1950 generation would have developed more normally but their ova would be vulnerable. Among the younger generation (1970–1989, 85% of post-closure pregnancy admissions), most were exposed from conception forward but were mainly unexposed during reproduction. Females exposed while ovaries are developing have damage to their reproductive system, which includes inhibition of the ability to conceive and when they do conceive, adverse pregnancy outcomes [60].

As compared with ROC, both generations of Willits women had significantly increased risk of pregnancy terminations and of bearing infants with abnormal weight or term, with no significant between-generation differences. Thus, as with animal studies, adverse outcomes were similar in both populations. The growing number of grandchildren will make possible the study of a third generation’s reproduction.

For now, the immediate question was how did babies fare? Since spontaneous abortion is the only condition studied in both animals and humans, we examined if the overall birth rate in Willits differed. In 1983, when the Plant had been using Cr(VI) for two decades, the Willits CBR was significantly lower than ROC. Compared with ROC and the State, the Willits CBR remained low through 2000, and did not rise to the State average until 12 years after Plant closure.

For the infant analysis, we focused on pre- and post-closure. From Table 1, most pre-closure births are births of the younger generation (1970–1989), exposed from conception forward, now having children post-closure. Health of post-closure babies, theoretically always unexposed, reflects the impact on reproductive capacity of early maternal exposure to Cr(VI).

In both infant and maternal models, regardless of time measure, and consistent with the established effect of Cr(VI), risk for abnormal birth weight or term was higher for Willits than ROC. Abnormal birth weight is often caused by endocrine/metabolic disorders in the mother [61–63]. During pregnancy and over the available life course, Willits women had elevated risk for endocrine conditions including thyroid disorders, diabetes, hyperlipidemia, and other endocrine conditions. Cr(VI) is a well-known endocrine disrupter [35].

Infants born before closure, primarily the 1970 generation of children born to the 1950 generation of mothers, were exposed to Cr(VI) in utero. Compared to ROC, that generation had significantly higher risk for a group of eye, ear, face, neck, cleft, and chromosomal anomalies and elevated risk for genitourinary anomalies. These are well-established effects of Cr(VI) exposure [23–25]. Post-closure, risk for both anomaly types dropped significantly.

We took particular interest in pregnancy admissions that resulted in termination without a live birth. Regardless of generation, pregnant women in Willits had significant risk of pregnancy loss, which we see in-hospital through ectopic pregnancies, spontaneous abortions, and therapeutic abortion procedures.

Ectopic pregnancies were the most frequent cause of in-hospital pregnancy loss in the younger generation of women, exposed during childhood. In the general population, these usually reflect defective embryo implantation due to scarred fallopian tubes caused by infectious pelvic inflammatory disease [64]. Cr(VI) is adept at producing non-infectious inflammation [65]. As reported earlier [21] and found here, Willits residents had increased risk of in-hospital screening for infections. This suggests that physicians, unaware of the effects of Cr(VI), struggle to find a septic etiology for conditions associated with inflammation.

Until closure, most Willits females were continuously exposed to Cr(VI). Subtle abnormalities may not become apparent until reproductive age, manifesting as sterility or miscarriage, bearing infants of abnormal weight or term, or having reproductive organ abnormalities. Compared to ROC, over the available life course, both generations of Willits females had increased risk for endometriosis, menstrual irregularities, ovarian cysts, and surgical procedures such as hysterectomies and oophorectomies. We also found higher cancer risk in both generations of Willits males and females, and among females, reproductive and benign neoplasms, predicting increased future risk in these and other cancers in the Willits population.

Unlike men whose spermatozoa can be damaged at any time from puberty onward, the female oocyte is particularly vulnerable to toxic exposure mutations at two periods: the first 13 weeks of gestation, and later as an adult at each ovulation. In the first weeks of female gestation, developing oocytes and supporting cells such as somatic granulosa and the extracellular matrix undergo maturational events. These primordial follicles lie dormant until puberty, when the ovary releases one or more monthly to begin follicle development. The oocyte once more becomes vulnerable to mutational change. These events during fetal development determine adult fertility and reproductive capacity.

Studies of pregnancies of exposed women cannot ignore the contribution of exposed mates. One of several articles reporting male mediated spontaneous abortion examined couples with metal worker husbands, planning their first pregnancy [36]. Loss increased linearly with the number of years men were exposed to Cr(VI). Animal studies confirm poor sperm quality of exposed males and fetal resorption when mated with unexposed females. This suggests peri-implantation mortality of fertilized ova, which certainly can contribute to low birth rate and spontaneous abortions, since many women would have married men who worked at the Plant, the largest employer in Willits. However, as others have reported in animal studies, reproductive problems in women exposed to Cr(VI) go beyond miscarriage.

In terms of general health, respiratory conditions are among the most firmly established adverse events for Cr(VI) exposure. In the newborn infant, in utero hypoxia/asphyxia is due to relative hypoxia of the mother. Respiratory distress in infants likely was caused by ambient Cr(VI). Before Plant closure, risk of these conditions were not different from ROC. Both dropped significantly post-closure in Willits infants.

When pregnant, women living in Willits had elevated risk of respiratory problems. When not pregnant, they had increased risk overall and for all sub-conditions with no generation difference. Local physicians had blamed high asthma rates on traditional air quality factors such as smog, smoke, wildfires, or burning trash. We earlier showed that these factors were not different between ROC and Willits [21]. The County was equal to or better than the State, and air quality as usually understood could not cause high illness rates in Willits. A recent California study found that the County has the third lowest lifetime asthma incidence in the State [66]. With similar results for males, our findings are consistent with established pulmonary scarring associated with Cr(VI) [67, 68].

Digestive system disorders and particularly liver disorders are other established sequelae of Cr(VI) exposure. Compared to ROC, Willits infants born of the 1950 generation had increased perinatal jaundice. This dropped significantly but remained elevated in infants of the 1970 Willits generation who became pregnant after Plant closure. The older generation of pregnant Willits women had a significantly lower risk of digestive system disorders than the younger generation, but over the life course, both generations had elevated risk of all digestive sub-system disorders. Cr(VI) is a well-established hepatotoxin, producing apoptosis and oxidative stress in human liver cells, and interacting with the liver at all maturation phases [69].

Unlike other general health conditions, risk for Willits infants to have nervous system conditions was significantly high and did not drop post-closure. Whether pregnant or not, both generations of Willits females and males had more nervous system and mental health conditions. When not pregnant, nervous system sub-conditions at greater risk included epilepsy, migraines, and congenital nervous system conditions, all understood to have underlying genetic components.

Cr(VI) readily crosses the blood brain barrier and deposits in the human brain [70], causing brain alterations, with marked degeneration of the cerebral cortex [71]. Cr(VI) conclusively causes behavioural deficits that are relevant for humans [72]. Costa and Klein identified central nervous system toxicity of Cr(VI) in workers [54]. Their findings mirror ours in domestically exposed humans.

Finally, we turn attention to the study strength and weaknesses. Other health officers and researchers concluded that exposure to Cr(VI) was high when the Plant was open and that exposure estimates likely were low. The range of symptoms elevated among Willits women and their infants is consistent with the complex impact of Cr(VI). Since beginning this work in 2007, we have used four different census-based population estimates and several different designs, all with consistent results.

Disease classification was reasonably accurate. Coding rules specify that physicians only identify secondary diagnoses considered relevant to treating the patient’s principal illness. Physicians identify these conditions over the course of care, with listing finalized at discharge. Hospital records are less subject to recall bias because they are the basis for developing treatment plans and billing for care. AHRQ developed the CCS to classify standard health conditions and procedures to facilitate surveillance and outcome research.

From the 1970 through 2000 census, these areas were in California’s top ranges for measures of population stability, and were demographically similar [21]. The 2010 census reports a large population drop for Willits proper but no drop in the enclosing ZIP 95490. This suggests people moved from Willits proper to the larger area. Census questions asking where people lived 5 years earlier show that residents tended to move locally and our linked life course models reflect this.

In these models, people may move but we know where they lived when admitted to hospital. About 4% moved within County, and proportionately more Willits residents moved to somewhere in ROC after ATSDR confirmed Plant exposures. This is why we slightly modified the life course design for this paper. However, this modification returned no important change in risk differences between Willits and ROC.

Our design assumes that residence at discharge was applicable to earlier points in time. In the case of pregnancy, we do not know if the mother was born in the area she gave as residence or how long she lived there before or during pregnancy. In the case of fetal exposure, most conceptions probably occurred in the area the mother identified as her residence at delivery. Other research suggests that incorrect geographic assignment of mothers or infants would create a bias toward the null hypothesis, but would not reduce the differences found [73, 74].

As described earlier [21], available data does not permit us to identify Plant employees or family members. People who worked in Willits (at the Plant or otherwise) and lived elsewhere will be classified incorrectly. County residents never hospitalized while living there are not included. Although data suggest population stability, we do not know how long patients lived in the County, when they arrived, or when (or if) they left. We have no way to assess these limitations or overcome them.

Remy separately addressed other possible methodologic issues [75]. She showed that: time measures used are valid both conceptually and quantitatively; hospital coding variability does not distort results; number of admissions before living in the County do not differ; demographic or socioeconomic differences between Willits and ROC are not significant, and both tend to be different from the State. Given Remy’s report, life course methods used here better address missing SSNC and movement between geographic areas.

Despite a number of small design modifications in various reports over now ten years, none importantly changed our understanding of health outcomes in Willits. In the context of uncertainty about geography, period, duration, and extent of exposure, we have come to believe that the risk reported based on ZIP-code is conservative, and that the true risk is more toward the upper tail of the confidence intervals.

We once more urge a well-designed study to collect data about individuals who lived in Willits during childhood and the reproductive period.

## Conclusions

For the first time, available data suggests the detrimental effects of domestic Cr(VI) exposure on human reproductive health. We focused primarily on reproductive health of exposed females and their babies. Our findings closely mirror those of many investigators reporting on all phases of animal Cr(VI) studies.

## Additional files

Additional file 1: Selecting records and classifying conditions. Groupers used to select pregnancies, identify deliveries, and categorize pregnancy outcomes. Groupers used to select infants, identify births, and categorize infant-specific outcomes. CCS diagnosis and procedure groupers used to classify other conditions and outcomes. (PDF 65 kb)
Additional file 2: Results of models to study infant outcomes by place of residence and before and after Plant closure. (XLSX 65 kb)
Additional file 3: Results of models to study general health and conditions specific to pregnancy by place of residence and birth generation. (XLSX 207 kb)
Additional file 4: Results of life course models to study general health and reproductive system outcomes for non-pregnant females by place of residence and birth generation. (XLSX 118 kb)
Additional file 5: Results of life course models to study general health and reproductive system outcomes for males by place of residence and birth generation. (XLSX 236 kb)

## Abbreviations

AHRQ: Agency for Health Research and Quality
ATSDR: Agency for Toxic Substance and Disease Registry
BD: Breslow-Day chi-square test for homogeneity
CBR: Crude birth rate
CCS: Clinical Classification Software
CL: 95% confidence limit
CMH: Adjusted Cochran-Mantel-Haenzel chi-square test
Cr(VI): Hexavalent chromium
DX: ICD-9 Diagnosis code
DXCH: ICD-9 DX coded to Body System Level 1 of CCS
DXCL: ICD-9 DX coded to CCS Disease Condition Level 2
LBW: Low birth weight
LCL: Lower 95% confidence limit
OSHPD: Office of Statewide Health Planning and Development
PDD: Patient discharge data
POF: Premature ovarian failure
PX: ICD-9 Procedure code
PXCH: ICD-9 PX coded to Body System Level 1 of CCS
PXCL: ICD-9 PX coded to CCS Disease Condition Level 2
ROC: Rest of county
RR: Relative risk
SGA: Small for gestational age
SSNC: Social Security Number, encrypted to protect confidentiality
UCL: Upper 95% confidence limit
