# Supplementary material for: Reproductive outcomes after non-occupational exposure to hexavalent chromium, Willits California, 1983-2014
Source: Environ Health. 2017 Mar 6;16:18. doi: 10.1186/s12940-017-0222-8 (PMC5340004; doi:10.1186/s12940-017-0222-8)
Supplement: Additional file 1: — Selecting records and classifying conditions. Groupers used to select pregnancies, identify deliveries, and categorize pregnancy outcomes. Groupers used to select infants, identify births, and categorize infant-specific outcomes. CCS diagnosis and procedure groupers used to classify other conditions and outcomes. (PDF 65 kb) [file 12940_2017_222_MOESM1_ESM.pdf]

Table 1: Groupers used to select pregnancies, identify deliveries, and categorize outcomes

| Grouper | Conditions |
|---|---|
| **MDC 14 Pregnancy Diagnosis-related groups 1983-2007** | 370 Cesarean section w cc; 371 Cesarean section w/o cc; 372 Vaginal delivery w complicating diagnoses; 373 Vaginal delivery w/o complicating diagnoses; 374 Vaginal delivery w sterilization &/or D&C; 375 Vaginal delivery w OR proc except sterilization &/or D&C; 376 Postpartum & post abortion diagnoses w/o OR procedure; 377 Postpartum & post abortion diagnoses w OR procedure; 378 Ectopic pregnancy; 379 Threatened abortion; 380 Abortion w/o D&C; 381 Abortion w D&C, aspiration curettage or hysterotomy; 382 False labor; 383 Other antepartum diagnoses w medical complications; 384 Other antepartum diagnoses w/o medical complications |
| **MDC 14 Pregnancy Diagnosis-related groups 2008-2012** | 765 Cesarean section w cc/mcc; 766 Cesarean section w/o cc/mcc; 767 Vaginal delivery w sterilization &/or D&C; 768 Vaginal delivery w OR proc except sterilization &/or D&C; 769 Postpartum & post abortion diagnoses w OR procedure; 770 Abortion w D&C, aspiration curettage or hysterotomy; 774 Vaginal Delivery w complicating diagnoses; 775 Vaginal delivery w/o complicating diagnoses; 776 Postpartum & post abortion diagnoses w OR procedure; 777 Ectopic pregnancy; 778 Threatened abortion; 779 Abortion w/o D&C; 780 False labor; 781 Other antepartum diagnoses w medical complications; 782 Other antepartum diagnoses w/o medical complications |
| **DRGs to identify delivery, used as denominator in some models** | **1983-2007**: 370 Cesarean section w cc; 371 Cesarean section w/o cc; 372 Vaginal delivery w complicating diagnoses; 373 Vaginal delivery w/o complicating diagnoses; 374 Vaginal delivery w sterilization &/or D&C; 375 Vaginal delivery w OR proc except sterilization &/or D&C |
| | **2008-2012**: 765 Cesarean section w cc/mcc; 766 Cesarean section w/o cc/mcc; 767 Vaginal delivery w sterilization &/or D&C; 768 Vaginal delivery w OR proc except sterilization &/or D&C; |
| **CCS Diagnoses** <br> **11: Conditions of pregnancy, birth, and puerperium** | 176 Contraceptives; 177 Spontaneous abortion; 178 Induced abortion; 179 Abortion complications; 180 Ectopic pregnancy; 181 Other pregnancy complications; 182 Hemorrhage during pregnancy; 183 Hypertension in pregnancy; 184 Early labor; 185 Long pregnancy; 186 Diabetes mellitus in pregnancy; 187 Malposition; 188 Pelvic obstruction; 189 Previous Cesarean section; 190 Fetal distress; 191 Amniotic cavity; 192 Umbilical cord; 193 OB-related perineal trauma; 194 Forceps delivery; 195 Other complications of birth; 196 Normal pregnancy/delivery |
| **CCS 181 Other pregnancy complications** | **Genitourinary infection in pregnancy (GUINFEC):** 64660 Gu Infect In Preg-Unspec; 64661 Gu Infection-Delivered; 64662 Gu Infection-Deliv W P/P; 64663 Gu Infection-Antepartum; 64664 Gu Infection-Postpartum |
| | **Fetal loss (FETLOSS):** 64600 Papyraceous Fetus-Unspec; 64601 Papyraceous Fetus-Deliv; 64603 Papyraceous Fet-Antepar; 64630 Habitual Aborter-Unspec; 64631 Habitual Aborter-Deliver; 64633 Habitual Abort-Antepart; 65130 Twins W Fetal Loss-Unsp (Begin 1989); 65131 Twins W Fetal Loss-Del (Begin 1989); 65133 Twins W Fetal Loss-Ante (Begin 1989); 65140 Triplets W Fet Loss-Unsp (Begin 1989); 65141 Triplets W Fet Loss-Del (Begin 1989); 65143 Triplets W Fet Loss-Ante (Begin 1989); 65150 Quads W Fetal Loss-Unsp (Begin 1989); 65151 Quads W Fetal Loss-Del (Begin 1989); 65153 Quads W Fetal Loss-Ante (Begin 1989); 65160 Mult Ges W Fet Loss-Unsp (Begin 1989); 65161 Mult Ges W Fet Loss-Del (Begin 1989); 65163 Mult Ges W Fet Loss-Ante (Begin 1989); 65640 Intrauterine Death-Unsp; 65641 Intrauter Death-Deliver; 65643 Intrauter Death-Antepart; V271 Deliver-Single Stillborn; V273 Del-Twins-1 Nb- 1 Sb; V274 Deliver-Twins- Both Sb; V276 Del-Mult Brth- Some Live; V277 Del-Mult Birth-All Sb; V2701 Deliver-Single Stillborn; V320 Twin- Mate Sb-In Hosp (Begin 1980 End 1989); V3200 Twin-Mate Sb-Hosp W/O Cs (Begin 1989); V3201 Twin-Mate Sb-Hosp W Cs (Begin 1989); V321 Twin- Mate Sb-Before Adm; V322 Twin- Mate Sb-Nonhosp; V350 Oth Multiple Sb-In Hosp (Begin 1980 End 1989); V3500 Oth Mult Sb-Hosp W/O Cs (Begin 1989); V3501 Oth Mult Sb-In Hosp W Cs (Begin 1989); V351 Oth Mult Sb-Before Adm; V352 Oth Multiple Sb-Nonhosp; V360 Multiple Nb/Sb-In Hosp (Begin 1980 End 1989); V3600 Mult Lb/Sb-In Hos W/O Cs (Begin 1989); V3601 Mult Lb/Sb-In Hosp W Cs (Begin 1989); V361 Mult Nb/Sb-Before Adm; V362 Multiple Nb/Sb-Nonhosp |
| **CCS 191 Amniotic cavity** | **Premature rupture Membrane (RUPTMEMB)**: ICD-9 DX 65810 Prem Rupt Membran-Unspec; 65811 Prem Rupt Membran-Deliv; 65813 Prem Rupt Memb-Antepart; 65820 Prolong Rupt Memb-Unspec; 65821 Prolong Rupt Memb-Deliv; 65823 Prolong Rup Memb-Antepar |
| | **Hydramnios (HYDRAMNIOS)**: ICD-9 DX 65700 Polyhydramnios-Unspec (Begin 1991); 65701 Polyhydramnios-Delivered (Begin 1991); 65703 Polyhydramnios-Antepart (Begin 1991); 65800 Oligohydramnios-Unspec; 65801 Oligohydramnios-Deliver; 65803 Oligohydramnios-Antepar |

| Grouper | Conditions |
|---|---|
| **CCS 195 Other complications of birth** | **Reproductive anomaly (REPROANOM):** ICD-9 DX 64850 Congen Cv Dis Preg-Unsp; 64851 Congen Cv Dis-Delivered; 64852 Congen Cv Dis-Del W P/P; 64853 Congen Cv Dis-Antepartum; 64854 Congen Cv Dis-Postpartum; 64930 Coagulation Def-Unspec (Begin 2006); 64931 Coagulation Def-Deliv (Begin 2006); 64932 Coagulatn Def-Del W P/P (Begin 2006); 64933 Coagulation Def-Antepart (Begin 2006); 64934 Coagulation Def-Postpart (Begin 2006); 64940 Epilepsy-Unspecified (Begin 2006); 64941 Epilepsy-Delivered (Begin 2006); 64942 Epilepsy-Delivered W P/P (Begin 2006); 64943 Epilepsy-Antepartum (Begin 2006); 64944 Epilepsy-Postpartum (Begin 2006); 64960 Uterine Size Descrp-Unsp (Begin 2006); 64961 Uterine Size Descrep-Del (Begin 2006); 64962 Uterine Size-Del W P/P (Begin 2006); 64963 Uterine Size Des-Antepar (Begin 2006); 64964 Uterine Size Descrep-P/P (Begin 2006); 64970 Cervical Shortening-Unsp (Begin 2008); 64971 Cervical Shortening-Del (Begin 2008); 64973 Cervical Shortening-Ante (Begin 2008); 65300 Pelvic Deform Nos-Unspec; 65301 Pelvic Deform Nos-Deliv; 65303 Pelv Deform Nos-Antepart; 65400 Cong Abn Uter Preg-Unsp; 65401 Congen Abn Uterus-Deliv; 65402 Cong Abn Uter-Del W P/P; 65403 Congen Abn Uter-Antepart; 65404 Congen Abn Uter-Postpart; 65430 Retrovert Uterus-Unspec; 65431 Retrovert Uterus-Deliver; 65432 Retrovert Uter-Del W P/P; 65433 Retrovert Uter-Antepart; 65434 Retrovert Uter-Postpart; 65440 Abn Grav Uterus Nec-Unsp; 65441 Abn Uterus Nec-Delivered; 65442 Abn Uterus Nec-Del W P/P; 65443 Abn Uterus Nec-Antepart; 65444 Abn Uterus Nec-Postpart; 65450 Cerv Incompet Preg-Unsp; 65451 Cervical Incompet-Deliv; 65452 Cerv Incompet-Del W P/P; 65453 Cerv Incompet-Antepartum; 65454 Cerv Incompet-Postpartum; 65460 Abn Cervix Nec Preg-Unsp; 65461 Abn Cervix Nec-Delivered; 65462 Abn Cervix Nec-Del W P/P; 65463 Abn Cervix Nec-Antepart; 65464 Abn Cervix Nec-Postpart; 65470 Abn Vagina In Preg-Unsp; 65471 Abnorm Vagina-Delivered; 65472 Abnorm Vagina-Del W P/P; 65473 Abnorm Vagina-Antepartum; 65474 Abnorm Vagina-Postpartum; 65480 Abn Vulva In Preg-Unspec; 65481 Abnormal Vulva-Delivered; 65482 Abnormal Vulva-Del W P/P; 65483 Abnormal Vulva-Antepart; 65484 Abnormal Vulva-Postpart; 65490 Abn Pel Nec In Preg-Unsp (Begin 1990); 65491 Abn Pelv Org Nec-Deliver (Begin 1990); 65492 Abn Pelv Nec-Deliv W P/P (Begin 1990); 65493 Abn Pelv Org Nec-Antepar (Begin 1990); 65494 Abn Pelv Org Nec-Postpar (Begin 1990); 65501 Fetal Cns Malform-Deliv; 65503 Fetal Cns Malfor-Antepar; 65510 Fetal Chromos Abn-Unspec; 65511 Fetal Chromoso Abn-Deliv; 65513 Fet Chromo Abn-Antepart; 65520 Famil Heredit Dis-Unspec; 65521 Famil Heredit Dis-Deliv; 65523 Famil Hered Dis-Antepart; 65530 Fet Damg D/T Virus-Unsp; 65580 Fetal Abnorm Nec-Unspec; 65581 Fetal Abnorm Nec-Deliver; 65583 Fetal Abnorm Nec-Antepar; 65590 Fetal Abnorm Nos-Unspec; 65591 Fetal Abnorm Nos-Deliver; 65593 Fetal Abnorm Nos-Antepar; OR DXCH14 (for mother)<br>**Large baby (FGROWTH)**. 65660 Excess Fetal Grth-Unspec; 65661 Excess Fetal Grth-Deliv; 65663 Excess Fet Grth-Antepart; |
| **CCS Procedures**<br>**12 Operations on the female genital organs** | 119 Oophorectomy; 120 Other ovary procedure; 121 Ligate fallopian tubes; 123 Other fallopian procedure ; 124 Hysterectomy; 125 Other excision of uterus; 126 Abortion; 127 Therapeutic D&C; 128 Diagnostic D&C; 129 Repair cystocele; 130 Other diagnosis female organ; 131 Non-operating room female genital organ; 132 Operating room female genital organ |
| **CCS Procedures**<br>**13: Obstetric procedures** | 122 Remove ectopic pregnancy; 133 Episiotomy; 134 Cesarean section; 135 Forceps breach; 136 Artificial rupture membrane; 137 Other assisted delivery; 138 Diagnostic amniocentesis; 139 Fetal monitoring; 140 OB laceration; 141 Other therapeutic OB procedure |

Table 2: Groupers used to select infants, identify births, and categorize outcomes

| Grouper | Conditions |
|---|---|
| **Infant** | Age less than one year at admission |
| **Newborn** | Infant and admission source was "Newborn" |
| **MDC 15 Newborn**<br>**Diagnosis-related groups 1983-2007** | 385 Neonates, died or transferred to another acute care facility; 386 Extreme immaturity or respiratory distress syndrome, neonate; 387 Prematurity w major problems; 388 Prematurity w/o major problems; 389 Full term neonate w major problems; 390 Neonate w other significant problems; 391 Normal newborn |
| **MDC 15 Newborn**<br>**Diagnosis-related groups 2008-2012** | 789 Neonates, died or transferred to another acute care facility; 790 Extreme immaturity or respiratory distress syndrome, neonate; 791 Prematurity w major problems; 792 Prematurity w/o major problems; 793 Full term neonate w major problems; 794 Neonate w other significant problems; 795 Normal newborn |
| **CCS Newborn Diagnoses**<br>**15 Conditions originating in the perinatal period** | 218 Liveborn; 219 Short gest, low wt, growth retard; 220 Birth asphyxia; 221 Respiratory distress; 222 Perinatal jaundice; 223 Birth trauma; 224 Other perinatal diagnosis |
| **CCS 224 Other perinatal diagnosis** | **Large for gestational age, macrosomia (LGAGE)**: ICD-9 DX 7660 Exceptionally Large Baby; 7661 Heavy-For-Date Infan Nec; 7662 Post-Term Infant Nos (End 2003) |
| **CCS Infant Diagnoses** | |
| **14 Congenital anomalies** | 213 Cardiac; 214 Gastrointestinal; 215 Genitourinary; 216 Nervous system; 217 Other congenital anomalies |
| **CCS 217 Other congenital anomalies** | **Eye, ear, face, neck, cleft (EEFNCANOM)**: ICD-9 DX 74310 Microphthalmos Nos; 74311 Simple Microphthalmos; 74312 Microphth W Oth Eye Anom; 74320 Buphthalmos Nos; 74321 Simple Buphthalmos; 74322 Buphthal W Oth Eye Anom; 74330 Congenital Cataract Nos; 74331 Capsular Cataract; 74332 Cortical/Zonular Catarac; 74333 Nuclear Cataract; 74334 Cong Tot/Subtot Cataract; 74335 Congenital Aphakia; 74336 Anomalies Of Lens Shape; 74337 Congenital Ectopic Lens; 74339 Cong Catar/Lens Anom Nec; 74341 Anom Corneal Size/Shape; 74342 Cong Cornea Opac Aff Vis; 74343 Cong Corneal Opacit Nec; 74344 Anom Anter Chamber-Eye; 74345 Aniridia; 74346 Anom Iris & Cil Body Nec; 74347 Anomalies Of Sclera; 74348 Mult Anom Anter Seg-Eye; 74349 Anom Anter Seg Nec-Eye; 74351 Vitreous Anomalies; 74352 Fundus Coloboma; 74353 Cong Chorioretinal Degen; 74354 Cong Fold/Cyst Post Eye; 74355 Cong Macular Change-Eye; 74356 Cong Retinal Changes Nec; 74357 Optic Disc Anomalies; 74358 Vascular Anom Post Eye; 74359 Post Segmnt Anom Nec-Eye; 74361 Congenital Ptosis; 74362 Congenital Eyelid Deform; 74363 Spec Anom Of Eyelid Nec; 74364 Spec Lacrimal Gland Anom; 74365 Spec Lacrimal Pass Anom; 74366 Spec Anomaly Of Orbit; 74369 Anom Eyelid/Lacr/Orb Nec; 74400 Ear Anom Nos/Impair Hear; 74401 Cong Absence Ext Ear; 74402 Ex Ear Anm Nec-Impr Hear; 74403 Middle Ear Anomaly Nec; 74404 Anomalies Ear Ossicles; 74405 Anomalies Of Inner Ear; 74409 Ear Anom Nec/Impair Hear; 74421 Cong Absence Of Ear Lobe; 74422 Macrotia; 74423 Microtia; 74424 Eustachian Tube Anom Nec; 74429 Ear Anomalies Nec; 74441 Branch Cleft Sinus/Fistu; 74442 Branchial Cleft Cyst; 74443 Cervical Auricle; 74446 Preauricular Sinus/Fistu; 74447 Preauricular Cyst; 74449 Branchial Cleft Anom Nec; 74481 Macrocheilia; 74482 Microcheilia; 74483 Macrostomia; 74484 Microstomia; 74489 Cong Face/Neck Anom Nec; 74900 Cleft Palate Nos; 74901 Unilat Cleft Palate-Comp; 74902 Unilat Cleft Palate-Inc; 74903 Bilat Cleft Palate-Compl; 74904 Bilat Cleft Palate-Inc; 74910 Cleft Lip Nos; 74911 Unilat Cleft Lip-Compl; 74912 Unilat Cleft Lip-Imcompl; 74913 Bilat Cleft Lip-Complete; 74914 Bilat Cleft Lip-Incompl; 74920 Cleft Palate & Lip Nos; 74921 Unil Cleft Palat/Lip-Com; 74922 Unil Cleft Palat/Lip-Inc; 74923 Bilat Clft Palat/Lip-Com; 74924 Bilat Clft Palat/Lip-Inc; 74925 Cleft Palate & Lip Nec;<br>**Chromosomal (CROMANOM)**: ICD-9-DX 7580 Down-S Syndrome; 7581 Patau-S Syndrome; 7582 Edward-S Syndrome; 7583 Autosomal Deletion Synd (End 2004); 7584 Balance Autosom Transloc; 7585 Autosomal Anomalies Nec; 7586 Gonadal Dysgenesis; 7587 Klinefelter-S Syndrome; 7588 Sex Chromosome Anom Nec (End 1996); 7589 Chromosome Anomaly Nos |

Table 3: CCS diagnosis groupers used to further classify general health outcomes

| Grouper | Conditions |
|---|---|
| **01 Infectious and parasitic diseases** | 01 Tuberculosis; 02 Septicemia (except in labor); 03 Bacterial infection, unspecified site; 04 Mycoses; 05 HIV infection; 06 Hepatitis; 07 Viral infection; 08 Other infections, including parasitic; 09 Sexually transmitted infections (not HIV hepatitis); 10 Immunization screening for infectious disease |
| **02 Neoplasms** | 11 Head/neck; 12 Esophageal; 13 Stomach; 14 Colon; 15 Rectum/anus; 16 Liver/ibd; 17 Pancreas; 18 GI/peritoneal; 19 Bronchial/lung; 20 Other respiratory; 21 Bone/ct; 22 Skin melanoma; 23 Non-epitheleal; 24 Breast; 25 Uterus; 26 Cervix; 27 Ovary; 28 Female genital; 29 Prostate; 30Testis; 31 Male genital; 32 Bladder; 33 Kidney/renal; 34 Urinary organ; 35 Brain/nervous system; 36 Thyroid; 37 Hodgkin's disease; 38 Non-Hodgkin's lymphoma; 39 Leukemias; 40 Multiple myeloma; 41 Other primary; 42 Secondary malignancy; 43 Malignant neoplasm; 44 Neoplasm unspecified; 45 Maintenance chemotherapy/radiation; 46 Benign uterine neoplasm; 47 Other benign neoplasm |
| | **Gastrointestinal (GICAN):** CCS 12-18 |
| | **Lung (LUNGCAN):** CCS 19-20 |
| | **Reproductive (REPROCA):** CCS 24-31 |
| | **Blood/lymph (BLOODCAN);** CCS 37-40 |
| | **Benign (BENIGN):** CCS 46-47 |
| **03 Endocrine; nutritional; and metabolic diseases and immunity disorders** | 48 Thyroid disorders; 49 Diabetes mellitus without complication; 5Diabetes mellitus with complications; 51 Other endocrine disorders; 52 Nutritional deficiencies; 53 Disorders lipid metabolism; 54 Gout other crystal arthropathies; 55 Fluid electrolyte disorders; 56 Cystic fibrosis; 57 Immunity disorders; 58 Other nutritional, endocrine, metabolic disorders |
| **04 Diseases of the blood and blood-forming organ** | 59 Anemia; 60 Acute posthemorrhagic anemia; 61 Sickle cell anemia; 62 Coagulation and hemorrhagic disorders; 63 Diseases of white blood cells; 64 Other hematologic conditions |
| **05 Mental Illness** | 650 Adjustment disorders; 651 Anxiety disorders; 652 Attention-deficit, conduct disruptive behavior disorders; 653 Delirium, dementia, amnestic, other cognitive disorders; 654 Developmental disorders; 655 Disorders diagnosed in infancy, childhood, adolescence; 656 Impulse control disorders NEC; 657 Mood disorders; 658 Personality disorders; 659 Schizophrenia other psychotic disorders; 660 Alcohol-related disorders; 661 Substance-related disorders; 662 Suicide intentional self-inflicted injury; 663 Screening history mental health/substance abuse; 67Miscellaneous disorders |
| **06 Diseases of the nervous system and sense organs** | 76 Meningitis (exc caused by TB STD); 77 Encephalitis (exc caused by TB STD); 78 Other CNS infection poliomyelitis; 79 Parkinsons disease; 80 Multiple sclerosis; 81 Other hereditary degenerative nervous system conditions; 82 Paralysis; 83 Epilepsy, convulsions; 84 Headache, including migraine; 85 Coma, stupor, brain damage; 86 Cataract; 87 Retinal detachments, defects, vascular occlusion, retinopathy; 88 Glaucoma; 89 Blindness vision defects; 90 Inflammation, infection eye (exc caused by TB STD); 91 Other eye disorders; 92 Otitis media related conditions; 93 Conditions associated with dizziness vertigo; 94 Other ear sense organ disorders; 95 Other nervous system disorders |
| **07 Diseases of the circulatory system** | 96 Heart valve disorders; 97 Peri-, endo-, myocarditis, cardiomyopathy (exc caused by TB STD); 98 Essential hypertension; 99 Hypertension with complications secondary hypertension; 100 Acute myocardial infarction; 101 Coronary atherosclerosis other heart disease; 0102 Nonspecific chest pain; 103 Pulmonary heart disease; 104 Other ill-defined heart disease; 105 Conduction disorders; 106 Cardiac dysrhythmias; 107 Cardiac arrest ventricular fibrillation; 108 Congestive heart failure, nonhypertensive; 109 Acute cerebrovascular disease; 110 Occlusion stenosis precerebral arteries; 111 Other ill-defined cerebrovascular disease; 112 Transient cerebral ischemia; 113 Late effects cerebrovascular disease; 114 Peripheral visceral atherosclerosis; 115 Aortic, peripheral, visceral artery aneurysms; 116 Aortic peripheral arterial embolism thrombosis; 117 Other circulatory disease; 118 Phlebitis, thrombophlebitis thromboembolism; 119 Varicose veins lower extremity; 120 Hemorrhoids; 121 Other diseases veins lymphatics |
| **08 Diseases of the respiratory system;** | 122 Pneumonia (exc caused by TB STD); 123 Influenza; 124 Acute chronic tonsillitis; 125 Acute bronchitis; 126 Other upper respiratory infections; 127 Chronic obstructive pulmonary disease bronchiectasis; 128 Asthma; 129 Aspiration pneumonitis, food/vomitus; 130 Pleurisy, pneumothorax, pulmonary collapse; 131 Respiratory failure, insufficiency, arrest (adult); 132 Lung disease due to external agents; 133 Other lower respiratory disease; 134 Other upper respiratory disease |
| **09 Diseases of the digestive system** | 135 Intestinal infection; 136 Disorders teeth jaw; 137 Diseases mouth, excluding dental; 138 Esophageal disorders; 139 Gastroduodenal ulcer (except hemorrhage); 140 Gastritis duodenitis; 141 Other disorders stomach duodenum; 142 Appendicitis other appendiceal conditions; 143 Abdominal hernia; 144 Regional enteritis ulcerative colitis; 145 Intestinal obstruction without hernia; 146 Diverticulosis diverticulitis; 147 Anal rectal conditions; 148 Peritonitis intestinal abscess; 149 Biliary tract disease; 151 Other liver diseases; 152 Pancreatic disorders (not diabetes); 153 Gastrointestinal hemorrhage; 154 Noninfectious gastroenteritis; 155 Other gastrointestinal disorders |

| Grouper | Conditions |
|---|---|
| **10 Diseases of the genitourinary system** | 156 Nephritis, nephrosis, renal sclerosis; 157 Acute unspecified renal failure; 158 Chronic renal failure; 159 Urinary tract infections; 160 Calculus urinary tract; 161 Other diseases kidney ureters; 162 Other diseases bladder urethra; 163 Genitourinary symptoms ill-defined conditions; 164 Hyperplasia prostate; 165 Inflammatory conditions male genital organs; 166 Other male genital disorders; 167 Nonmalignant breast conditions; 168 Inflammatory diseases female pelvic organs; 169 Endometriosis; 170 Prolapse female genital organs; 171 Menstrual disorders; 172 Ovarian cyst; 173 Menopausal disorders; 174 Female infertility; 175 Other female genital disorders |
| **12 Diseases of the skin and subcutaneous tissue** | 197 Skin subcutaneous tissue infections; 198 Other inflammatory condition skin; 199 Chronic ulcer skin; 2 Other skin disorders; 201 Infective arthritis osteomyelitis (exc caused by TB STD); 202 Rheumatoid arthritis related disease; 203 Osteoarthritis; 204 Other non-traumatic joint disorders; 205 Spondylosis, intervertebral disc disorders, other back problems; 206 Osteoporosis; 207 Pathological fracture; 208 Acquired foot deformities; 209 Other acquired deformities; 210 Systemic lupus erythematosus connective tissue disorders; 211 Other connective tissue disease; 212 Other bone disease musculoskeletal deformities |
| **13 Diseases of the musculoskeletal system and connective tissue** | 201 Infectious arthritis; 202 Rheumatic arthritis ; 203 Osteoarthritis ; 204 Other joint diagnosis ; 205 Back problem; 206 Osteoporosis; 207 Pathologic fracture; 208 Acquired foot defect; 209 Other acquired deformity; 0210 SLE; 211 Other connective tissue; 212 Other bone diagnosis |

Table 4: CCS procedure groupers used to further classify outcomes

| Grouper | Conditions |
|---|---|
| **01 Operations on the nervous system** | 001 Inc/exc CNS; 002 Extcrn shunt; 003 Laminectomy; 004 Dx spinaltap; 005 Inject spine; 006 Decomp nerve; 007 Ot Dx nerv; 008 Ot nOR nerv; 009 Ot OR nerv |
| **02 Operations on the endocrine system** | 010 Thyroidect; 011 Dx endocr; 012 Ot Rx endoc |
| **03 Operations on the eye** | 013 Corneal tran; 014 Glaucoma prs; 015 Cataract prs; 016 Rpr retina; 017 Retin les pr; 018 Dx eye; 019 Ot Rx eye; 020 Ot intraocul; 021 Ot extraocul; |
| **04 Operations on the ear** | 022 Tympanoplas; 023 Myringotomy; 024 Mastoidectom; 025 Dx procs ear; 026 Ot Rx ear pr |
| **05 Operations on the nose; mouth; and pharynx** | 027 Ctrl epistax; 028 Plst pr nose; 029 Dental procs; 030 Tonsillect; 031 Dx nose mout; 032 nOR nose mou; 033 OR nose mout |
| **06 Operations on the respiratory system** | 034 Tracheostomy; 035 Tracheoscopy; 036 Pneumonectom; 037 Bronchoscopy; 038 Dx prs lung; 039 Thoracentesi; 040 Dx respirat; 041 n-OR respira; 042 OR respirato |
| **07 Operations on the cardiovascular system** | 043 Heart valve; 044 CABG; 045 PTCA; 046 Thrombolysis; 047 Cardiac cath; 048 Pacemaker; 049 Ot OR heart; 050 Extrcor circ; 051 Endarterecto; 052 Aort resect; 053 Varic vein; 054 Ot vasc cath; 055 Peri vas byp; 056 Ot vas byp; 057 AV fistula; 058 Hemodialysis; 059 Vesl hed/nck; 060 Vessel leg; 061 Ot vessels; 062 Ot Dx CV prs; 063 n-OR CV prs |
| **08 Operations on the hemic and lymphatic system** | 064 Bon mar tran; 065 Bon mar bx; 066 Prs spleen; 067 Ot Rx hemic |
| **09 Operations on the digestive system** | 068 Inject esop; 069 Esoph dilat; 070 UGI endosc; 071 Gastrostomy; 072 Colostomy; 073 Ileostomy; 074 Gastrectomy; 075 Sm bowel res; 076 Colonoscopy; 077 Proctoscopy; 078 Colorec resc; 079 Rem coln les; 080 Appendectomy; 081 Hemorrh prs; 082 ERCP; 083 Bx liver; 084 Cholecystec; 085 Ing/fem hern; 086 Ot hern repr; 087 Laparoscopy; 088 Abd paracent; 089 Explor lap; 090 Exc perit ad; 091 Perit dialys; 092 Ot bowel Dx; 093 nOR UGI Rx; 094 Ot OR UGI Rx; 095 nOR LGI prcs; 096 OR LGI prcs; 097 Ot GI Dx prs; 098 nOR GI Rx pr; 099 OR GI Rx prs |
| **10 Operations on the urinary system** | 100 Urin endosco; 101 Transur rem; 102 Ureter cath; 103 Nephrotomy; 104 Nephrectomy; 105 Kidney trans; 106 GU incont pr; 107 Lithotrip ur; 108 Urine cath; 109 Prcs urethra; 110 Dx prc urin; 111 nOR Rx urin; 112 OR Rx urin |
| **11 Operations on the male genital organs** | 113 TURP; 114 Opn prostect; 115 Circumcision; 116 Dx male gen; 117 nOR male gen; 118 OR male gen |
| **12 Operations on the female genital organs** | 119 Oophorectomy; 120 Ot prc ovary; 121 Ligat fallop; 123 Ot prs fallo; 124 Hysterectomy; 125 Ot excis ut; 126 Abortion; 127 Rx D&C; 128 Dx D&C; 129 Rep cystocel; 130 Ot Dx female; 131 nOR female; 132 OR female |
| **14 Operations on the musculoskeletal system** | 142 Excis bone; 143 Bunionectomy; 144 Rx; 145 Rx fx radius; 146 Rx fx hip; 147 Rx fx leg; 148 Ot fx procs; 149 Arthroscopy; 150 Div joint; 151 Exc cart kne; 152 Arthropl kne; 153 Hip replace; 154 Ot arthropla; 155 Arthcentesis; 156 Inject musc; 157 Amputat leg; 158 Spinal fus; 159 Dx prs muscu; 160 Rx prs musc; 161 OR Rx bone; 162 OR Rx joints; 163 nOR muscskel; 164 OR muscskel |
| **15 Operations on the integumentary system** | 165 Breast bx; 166 Lumpectomy; 167 Mastectomy; 168 I&D skin; 169 Debrid wound; 170 Excis skin; 171 Suture skin; 172 Skin graft; 173 Ot Dx skin; 174 nOR Rx skin; 175 OR Rx skin |
